# Epidermal Growth Factor Receptor Mutations Carried in Extracellular Vesicle-Derived Cargo Mirror Disease Status in Metastatic Non-small Cell Lung Cancer

**DOI:** 10.3389/fcell.2021.724389

**Published:** 2021-10-06

**Authors:** Emma Purcell, Sarah Owen, Emily Prantzalos, Abigail Radomski, Nayri Carman, Ting-Wen Lo, Mina Zeinali, Chitra Subramanian, Nithya Ramnath, Sunitha Nagrath

**Affiliations:** ^1^Department of Chemical Engineering, University of Michigan, Ann Arbor, MI, United States; ^2^Biointerfaces Institute, University of Michigan, Ann Arbor, MI, United States; ^3^Department of Surgery, University of Michigan, Ann Arbor, MI, United States; ^4^Department of Internal Medicine, University of Michigan, Ann Arbor, MI, United States; ^5^Veterans Affairs Ann Arbor Healthcare System, Ann Arbor, MI, United States; ^6^Rogel Cancer Center, University of Michigan, Ann Arbor, MI, United States

**Keywords:** extracellular vesicle (EV), EGFR mutation, longitudinal monitoring, resistance mutation, non-small cell lung cancer, EV-protein, EV-RNA, tyrosine kinase inhibitor (TKI)

## Abstract

In non-small cell lung cancer (NSCLC), identifying the presence of sensitizing and resistance epidermal growth factor receptor (EGFR) mutations dictates treatment plans. Extracellular vesicles (EVs) are emerging as abundant, stable potential liquid biopsy targets that offer the potential to quantify EGFR mutations in NSCLC patients at the RNA and protein level at multiple points through treatment. In this study, we present a systematic approach for serial mutation profiling of 34 EV samples from 10 metastatic NSCLC patients with known EGFR mutations through treatment. Using western blot and droplet digital PCR (ddPCR), sensitizing (exon 19 deletion, L858R) mutations were detected in EV-Protein, and both sensitizing and resistance (T790M) mutations were quantified in EV-RNA. EGFR mutations were detected in EV-Protein from four patients at multiple time points through treatment. Using EV-RNA, tumor biopsy matched sensitizing mutations were detected in 90% of patients and resistance mutations in 100% of patients. Finally, mutation burden in EV-RNA at each time point was compared to disease status, described as either stable or progressing. For 6/7 patients who were longitudinally monitored through treatment, EV mutation burden mirrored clinical trajectory. When comparing mutation detection between EV-RNA and ctDNA using ddPCR, EVs had a better detection rate for exon 19 deletions and the L858R point mutation. In conclusion, this study demonstrates that integrating EV analysis into liquid biopsy mutation screening has the potential to advance beyond the current standard of care “rule in” test. The multi-analyte testing allows future integration of EGFR mutation monitoring with additional EV-markers for a comprehensive patient monitoring biomarker.

## Graphical Abstract

EVs are isolated from plasma collected from NSCLC patients harboring known EGFR mutations using ultracentrifugation. RNA and protein are extracted from the EV samples and tested for EGFR mutations using ddPCR and western blot, respectively.

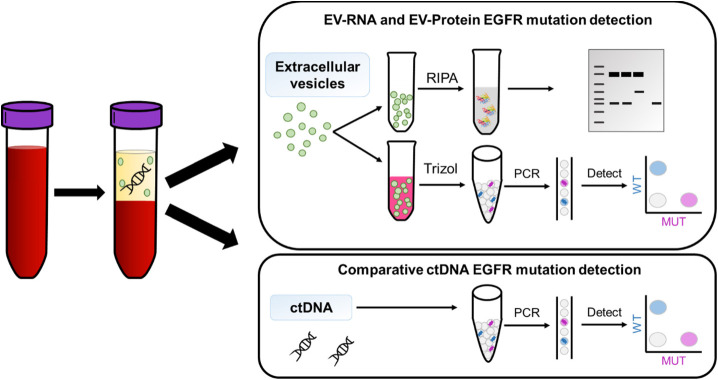

## Introduction

The widespread adoption of targeted therapies using small molecule tyrosine kinase inhibitors (TKIs) has greatly benefited the 10–30% of advanced non-small cell lung cancer (NSCLC) patients who have sensitizing epidermal growth factor receptor (EGFR) mutations in an otherwise challenging to treat cancer (Collisson et al., 2014; Zhang et al., 2016). This subset of NSCLC patients harboring sensitizing [L858R and exon 19 deletion (exon 19 del)] EGFR mutations have seen significantly improved survival due to TKIs; yet resistance often occurs in as few as 9 months, commonly through the secondary EGFR T790M mutation (Clark et al., 2005; Pao et al., 2005; Balak et al., 2006). While it has been documented that additional mutations arise during TKI treatment, traditional tumor monitoring technologies are not commonly used to monitor for these changes. To improve patient care, it is critical to have real-time knowledge of a patient’s mutation status, thereby allowing clinicians to alter treatment strategies accordingly. As such, the development of a method for non-invasive longitudinal monitoring offers the potential to drastically improve real-time treatment personalization.

Due to the invasiveness and inherently localized sampling of tissue biopsies, they are not frequently used for repeated monitoring, may not result in enough material for testing, and may miss mutations carried in other tumor regions [National Comprehensive Cancer Network (NCCN)/NCCN Foundation, 2021]. To address these challenges, advancements in liquid biopsies have led to the clinical use of blood-based biomarkers, mostly commonly circulating tumor DNA (ctDNA), to monitor changes in the tumor non-invasively and longitudinally (Alix-Panabières and Pantel, 2016). Yet, ctDNA assays are limited by several notable technical challenges; ctDNA is shed only during cell death (Jahr et al., 2001) and suffers from low abundance (Diehl et al., 2005) and rapid clearance from circulation (Gauthier et al., 1996; Kustanovich et al., 2019). Hence, ctDNA has the potential to miss the most current molecular changes in tumor cells that are the most evasive leading to treatment resistant.

Extracellular vesicles (EVs), lipid bilayer bound nanovesicles approximately 30–150 nm in diameter, are a promising alternative blood-based biomarker offering increased abundance and stability compared to ctDNA (Jin et al., 2016; Kang et al., 2016). They are functional vesicles secreted from live cells as a mechanism of cell-cell communication and contain cargo from their originating cells, including DNA, RNA, and protein, which is protected by the lipid bilayer from exogenous degradation while in circulation (Théry, 2011). The stability of these vesicles in circulation is the key distinguishing factor compared to ctDNA.

Given the tiny amount of cargo carried by these nano-sized vesicles, the development of techniques that can analyze these important carriers is an active area of research. To date, research has largely been limited to microRNA or protein to glean information about a patient’s disease, and to develop diagnostic and prognostic signatures (Rabinowits et al., 2009; Lobb et al., 2017). A final key benefit of using EVs for a liquid biopsy compared to ctDNA is the ability to perform multiplexed analysis of EV-derived RNA (EV-RNA) and EV-derived protein (EV-protein). However, thus far, the detection of mutations carried in EVs in either analyte has been reported by few groups and has been biased toward nucleic acid analysis, with few groups reporting the detection of mutant proteins in extracellular vesicles (Lobb et al., 2017).

Significantly, mutation profiling in extracellular vesicles is an emerging field, with the first studies focusing on the detection of cancer-specific mutations (Chen et al., 2013; Thakur et al., 2014; Figueroa et al., 2017). Mutations carried in EVs have been shown in glioblastoma, pancreatic cancer, and NSCLC. The clinical application has centered on improving detection rates compared to ctDNA alone, commonly by combining ctDNA with DNA and RNA derived from exosomes (a subset of small EVs) (Castellanos-Rizaldos et al., 2018; Krug et al., 2018). For example, Castellanos-Rizaldos et al. (2018) co-isolated exosomal RNA and cell-free RNA for mutation detection from a panel of 30 EGFR mutations, achieving a higher sensitivity than with cell-free RNA alone. Similar to other studies, this work was limited based on the use of pre-amplification steps prior to qPCR, which can introduce signal strength bias, for mutation detection and was constrained to a single time point for evaluation. While increasing the detection rate of these rare mutations was achieved, these studies have not demonstrated their utility in a clinical setting and remain single-analyte analyses.

Several recent works have demonstrated the feasibility of using EV-RNA to detect EGFR mutations from blood (Dong et al., 2019; Pasini et al., 2021) and bronchoalveolar lavage fluid (Hur et al., 2019; Liam et al., 2020). Notably, a robust method was developed for detecting mutations in EV-RNA using droplet digital PCR (ddPCR) and was validated with Sanger sequencing with mutations found with over 90% sensitivity. The study profiled EVs before treatment and at the time of progression events in a cohort of metastatic NSCLC patients, further demonstrating the promise of using EV-RNA to monitor mutations through treatment (Pasini et al., 2021). There remains, however, a need to verify whether changes in mutation burden, at either the RNA or protein level, found within EVs, is a predictor of progression events as opposed to a result of disease progression.

The study presented here builds upon these previous works by following a cohort of metastatic NSCLC patients to quantify the EGFR mutation burden carried in both EV-RNA and EV-Protein. Specifically, ddPCR was used to analyze the EV-RNA without pre-amplification while mutated protein content of EVs was profiled using western blot. Patients carried at least one sensitizing EGFR mutation (exon 19 del, L858R) based on tumor biopsy and were receiving TKI therapy at the time of enrollment. Moreover, a subset of patients in this study already carried the resistance T790M mutation at the beginning of longitudinal monitoring. Resistance mutations would potentially decrease the efficacy of TKIs, although novel TKIs are being developed to target these mutations as well. Despite the predicted TKI sensitivity, patients had differing therapeutic responses, which is a well-known challenge of TKI therapies. As such, there is a need to develop an enhanced approach to predict who will have favorable outcomes, or who should receive an alternative therapy.

To this end, in this study the cohort was longitudinally monitored for EGFR mutation burden carried by EV-RNA and EV-protein. This study expanded upon previous works by monitoring patients serially, allowing for time points before, during, and after progression events. Finally, expanding beyond only EV-RNA profiling highlights the potential benefits of dual-monitoring both EV-RNA and EV-protein; distinct roles for EV-RNA and EV-protein were revealed.

## Results

### Extracellular Vesicle Cargo Carries Mutations From Cells of Origin

To first establish experimental protocols and demonstrate the presence of EGFR mutations in EVs, EVs secreted from lung cancer cell lines with known EGFR mutations were tested for EGFR mutations. EV-RNA and EV-protein were tested using ddPCR and western blot, respectively. ddPCR offers a highly sensitive platform to directly quantify the number of mutant transcripts from bulk RNA without risk of pre-amplification bias (Owen et al., 2020), allowing for direct comparison between samples and patients. Briefly, the EVs were lysed using TRIzol^TM^ Reagent. The Norgen Single Cell RNA kit (Norgen Biotek Corp.) was used to purify and isolate the EV-RNA, due to the miniscule amount of RNA contained in these samples. RNA was reverse transcribed and directly loaded onto the RainDrop^TM^ (RainDance Technologies) ddPCR system for analysis.

**FIGURE 1:**
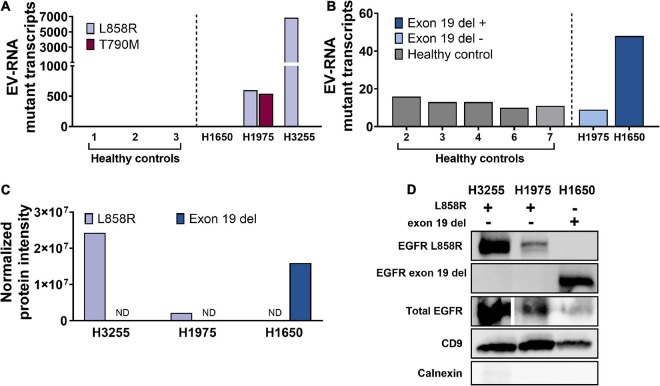
EGFR mutations carried in RNA and protein from cell line derived EVs H1975 (L858R/T790M), H3255 (L858R), H1650 (exon 19 del). **(A,B)** EV-RNA ddPCR droplet counts of lung cancer cell line-derived and healthy plasma for **(A)** L858R and T790M point mutations and **(B)** exon 19 del. **(C)** Normalized protein intensity for L858R and exon 19 del EGFR mutations from cell line derived extracellular vesicles using western blots. Normalized protein intensity was calculated using Bio-Rad’s Stain Free Blot technology to compare specific bands to the total protein of each lane. **(D)** Western blot of cell line derived EVs tested for L858R, exon 19 del, total EGFR, CD9, and calnexin. ND, not detected.

Matching the cells of origin, EV-RNA derived from H3255 carried a heterozygous L858R mutation (Figure 1A and Supplementary Figure 1), EVs from H1975 carried heterozygous L858R and T790M mutations (Figure 1A), and EVs from H1650 carried exon 19 del (Figure 1B). As expected, the assay showed that the EVs were negative for EGFR mutations that were not present originally in their cell line of origin (Figures 1A,B). In healthy donor EV-RNA (1–3), there were 0.33 ± 0.47 (*n* = 3) L858R droplets and 0 ± 0 (*n* = 3) T790M droplets (Figures 1A,B and Supplementary Figure 2). However, when assaying healthy controls, the exon 19 del ddPCR assay showed an average background of 12.6 ± 2 (*n* = 5) droplets in healthy donor EV-RNA, similar to the background signal from the negative control cell line EV-RNA H1975. This assay simultaneously screens for 19 different deletion variants using pooled primers, resulting in increased background signal. A threshold for positive detection was determined based on the highest background signal observed among all negative control samples. This threshold for detection was used for all subsequent analysis for NSCLC patient EV-RNA.

Cell line derived EVs also carried mutant EGFR protein, as demonstrated using western blot. Specific identification of the two activating mutant proteins was achieved. L858R was detected exclusively in H3255 and H1975 derived EVs (Figures 1C,D), and exon 19 del only in H1650 derived EVs (Figure 1D). A validated T790M antibody is not yet commercially available, and therefore the samples were not tested for this mutation. Additionally, the cell line derived EVs were tested for total EGFR abundance probing for a conserved, wildtype region of EGFR (Figure 1D). Each EV-protein sample was additionally profiled for the EV marker CD9, and was shown to be free of cellular contamination based on calnexin (Figure 1D).

Protein intensity for each marker was normalized to the total protein loaded into each lane using Bio-Rad’s Stain Free Gel technology. Each band was normalized to the total protein per lane, eliminating the need for housekeeping genes which are not equally present in all EVs due to their loading mechanisms and cells of origin (Théry et al., 2018). Stain Free Gels have been found to be more consistent than housekeeping proteins or Ponceau staining as a loading control and provide the added benefit of controlling for differential loading (Gilda and Gomes, 2013; Rivero-Gutiérrez et al., 2014). The samples were loaded based on a normalized starting cell number and EV biogenesis time, 3 million cells for 72 h incubation, which, for perspective was quantified to be 7.5 μg for H3255 EVs, 3.5 μg for H1975 EVs, and 3.1 μg for H1650 EVs.

### Metastatic Non-small Cell Lung Cancer Patient Cohort and Study Design for Epidermal Growth Factor Receptor Mutational Profiling in Extracellular Vesicles

The above established dual EV-RNA and EV-protein mutational profiling was applied to analyze EVs from blood plasma in a cohort of ten metastatic NSCLC patients with at least one known sensitizing EGFR mutation based on primary tumor biopsy. Patients were enrolled after consent and blood was collected under IRB approval. The cohort’s median age was 64 years (range, 45–82 years) and was well distributed between male and female. Full patient demographics can be found in Supplementary Table 1.

Seven of the ten patients had samples collected at multiple time points, termed visits. The time between each visit varied depending on patient care, however, the time ranged from 26 to 231 days (mean = 89 ± 40 days) across all patients. The visit notation is used throughout this study to highlight general trends on the utility of patient monitoring using EVs to be compared across patients, however, patient specific details and timelines are shown in Supplementary Figure 3 and Supplementary Table 1. The blood samples were preprocessed to isolate plasma using red blood cell depletion methods, validation experiments demonstrating this material can be utilized for EV analysis are shown in Supplementary Figure 2.

While a plethora of EV isolation methods have been widely developed, including ultracentrifugation (Théry et al., 2006), microfluidic devices for EV capture (Kanwar et al., 2014; Kang et al., 2019), and commercially available kits such as ExoQuick (System Biosciences), EVs in this study were isolated using ultracentrifugation, which offers the widest array of downstream applications and high purity compared to the above methods (Tang et al., 2017; Patel et al., 2019) and is compatible with a range of sample input volumes. After isolation, EV concentration and size was determined using nanoparticle tracking analysis (NTA), Supplementary Table 2. Each EV sample was tested for EGFR mutations matching the tumor biopsy result. Additionally, matching ctDNA samples, nine samples across five patients, were tested for the corresponding EGFR mutations to compare detection rates.

### Longitudinal Detection of Epidermal Growth Factor Receptor Mutations in EV-Protein

**FIGURE 2:**
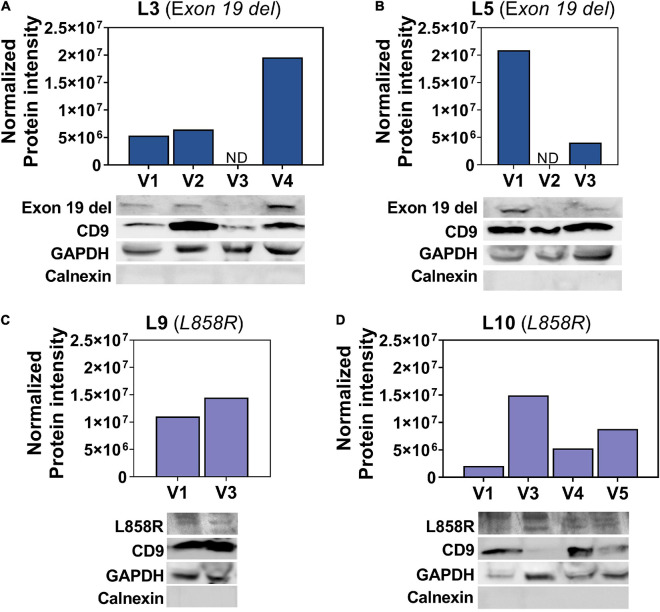
Detection of EGFR mutations in EV-protein. **(A–D)** EV-protein with detected mutant EGFR from patients across multiple visits from **(A)** L3 with exon 19 del, **(B)** L5 with exon 19 del, **(C)** L9 with L858R, and **(D)** L10 with L858R. Samples were additionally screened for CD9, GAPDH, and calnexin. Normalized mutant EV-protein is quantified above each western blot using Bio-Rad’s Stain Free Gel technology to normalize to the total protein as quantified by imaging the Stain Free Gel after transfer to a PVDF membrane. ND, not detected.

EV-protein was isolated from the plasma of four patients over 2–4 visits. EV-protein was tested for sensitizing mutations exon 19 del or L858R based on initial biopsy. EGFR mutations in the EV-protein were detected using western blot in samples from 4/4 (100%) patients, two patients with exon 19 del mutations and two with L858R mutations. Patients L3 and L5 have moderately identifiable bands for exon 19 del, while having various levels of CD9 and similar GAPDH bands, Figures 2A,B. The two patients demonstrated similar detection rates, with one patient having exon 19 del EV-protein detected in 3/4 of samples (L3) and the second in 2/3 of samples (L5). Interestingly, one patient has a trending increase in their mutated EGFR EV-protein, while the other has a steady decrease, despite both being clinically stable through all time points.

Conversely, the L858R band is present in samples from patients L9 and L10, but less distinct compared to exon 19 del in L3 and L5, Figures 2C,D. While the bands are less distinct, there is L858R EV-protein at all the time points tested. The second patient, L10, shows a net increase in L858R EV-protein over time compared to visit one, although the amount does not increase at all visits. The less optimal bands could be the result of EV packaging, the protein itself, or several other challenges. However, the CD9 and GAPDH proteins are highly variable between samples for the patients with L858R and are less clean than would be expected from either cells or healthy EVs.

Epidermal growth factor receptor mutations found in EV-protein demonstrate the potential to use EVs as multiple cargo biomarkers. This finding marks the first demonstration of EGFR mutations detected in patient-derived EV-protein. Full western blots can be found in Supplementary Figure 4.

### Epidermal Growth Factor Receptor Mutations in EV-RNA Detected in Metastatic Non-small Cell Lung Cancer Patients

**FIGURE 3:**
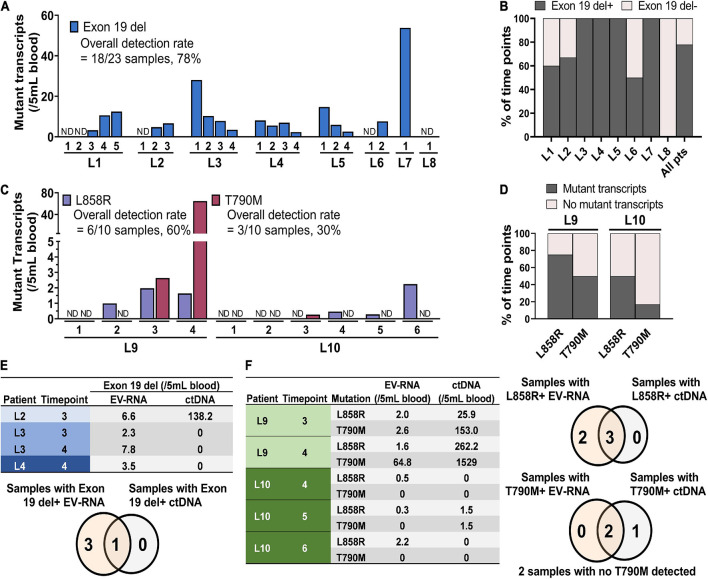
Detection of EGFR mutations in EV-RNA samples from metastatic NSCLC patients. **(A)** EGFR exon 19 del transcript concentration per time point for the eight patients pre-identified as being exon 19 del positive. **(B)** Percent of time points that tested positive for EGFR exon 19 del mutations per patient. **(C)** Mutant EV-RNA concentration for both L858R and T790M mutations across time points for two patients. **(D)** Percent of time points that tested positive for L858R and T790M mutations by patient. **(E,F)** Comparative concentration of EGFR mutations found in ctDNA and EV-RNA along with Venn diagrams displaying the overlap in samples with detected mutations in EV-RNA and ctDNA for **(E)** four samples with known exon 19 del mutations and **(F)** five samples with known L858R and T790M mutations.

In compliment, EV-RNA was isolated from 10 patients with multiple samples (*n* = 33 total samples) collected from each of these patients at different time points (up to six visits) through their course of treatment. The mutation burden was evaluated in each sample, defined as the number of mutant EGFR droplets detected using ddPCR normalized to 5 mL of starting blood volume. Exon 19 del was detected in 7/8 patients, Figure 3A. Patients varied as to the number of time points with exon 19 del positive EV-RNA, with some patients having mutant EV-RNA at 100% of time points (L3, L4, L5, and L7), and one having no detected exon 19 del, L8, Figure 3B. It is important to note that the patients had varying numbers of visits, ranging from 1 to 5 (average = 2.88, *n* = 8). Taken together, the positivity rate across all samples for exon 19 del was 78% (*n* = 18/23).

The remaining two patients L9 and L10 co-harbored L858R and T790M mutations. Although the data is collected from only two patients, there was a larger range in the number of mutant transcripts detected for the two point mutations than were observed for exon 19 del. Patient L9 had a range of 0–80 T790M transcripts detected, compared to L10 with a range from 0 to 0.5 transcripts per 5 mL blood, Figure 3C. Additionally, the point mutations were detected less frequently per patient compared to the patients with exon 19 del. L858R was detected in 75% of time points in L9 and in 50% of time points in L10. Even more modest was the detection of T790M, found in 50% of visits in L9 and only 17% of visits in L10, Figure 3D.

For nine samples, matched ctDNA was tested alongside EV-RNA for the EGFR mutations using the same ddPCR technique. For patients with exon 19 del, ctDNA was found in only 1/4 samples tested, compared to 4/4 EV-RNA samples, Figure 3E. Similarly, five L858R/T790M samples had dual testing and results show that 3/5 samples tested positive for L858R in ctDNA, whereas 5/5 samples were positive for L858R in EV-RNA. The T790M was detected less frequently in both ctDNA and EV-RNA, with only 2/5 samples having mutations in both ctDNA and EV-RNA, one having only ctDNA mutations, and two having no detected T790M mutations, Figure 3F.

Finally, mutation burden between EV-RNA, EV-Protein, and ctDNA were analyzed to determine if there was a correlation between any of the three factors. Using a simple linear regression, it was found that the concentration of mutation burdens between the analytes (EV-RNA, EV-Protein, and ctDNA) were not correlated based off their *R*^2^ values for the three mutations (exon 19 del, L858R, and T790M), Supplementary Figure 5.

### Longitudinal Monitoring of Epidermal Growth Factor Receptor Mutations in EV-RNA Mirrors Disease Trajectory

**FIGURE 4:**
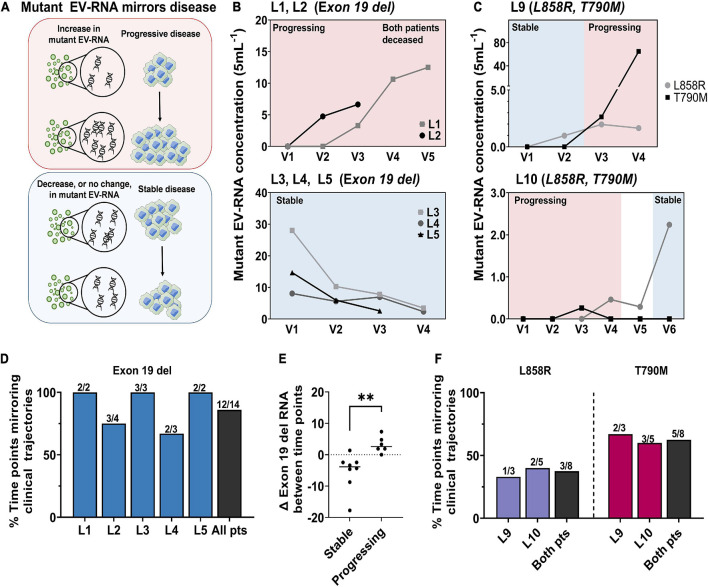
Changes in EGFR mutation burden in EV-RNA mirror disease status. **(A)** Schematic demonstrating EV-RNA mirroring disease status. An increase in mutant EV-RNA mirrors progressive disease, while a decrease or no change in EV-RNA would mirror stable disease. **(B)** Mutant EV-RNA concentration for exon 19 del patients across multiple visits for patients with consistently progressive (top) or consistently stable (bottom) disease. **(C)** Mutant EV-RNA concentration for L858R/T790M in divergent patients, L9 (top) and L10 (bottom) across multiple visits. **(D)** Percent of EV-RNA samples drawn at a time point when EV-RNA mutation burden mirrors disease status for patients with exon 19 del. **(E)** Change (Δ) exon 19 deletion mutation burden in EV-RNA between time points for patients who are clinically stable compared to progressing, *p*-value = 0.0059 using an unpaired *t*-test. **(F)** Percent of EV-RNA samples drawn at a time point when EV-RNA mutation burden mirrors disease status for patients with L858R and T790M mutations. **p-value ≤ 0.01.

To explore the utility of EV-RNA in patient care, transient mutant EGFR burden was compared to the clinical outcomes of seven patients across up to six visits, Supplementary Figure 3. At each time point, response to therapy is classified as either a stable (*n* = 17 samples) or progressing (*n* = 22 samples) based on available clinical data corresponding to each blood draw visit following the guidelines established in Response Evaluation Criteria in Solid Tumors (RECIST; Eisenhauer et al., 2009). EV-RNA was determined to mirror disease status when an increase in mutation burden occurred at the same time point as progression, or conversely if a decrease or no change in mutation burden occurred at a time point when a patient was determined to have stable disease, Figure 4A.

Each patient’s progression was classified based on overall disease trajectory into one of three categories based on trend in EV-RNA burden over time: consistently progressing (*n* = 2 patients) Figure 4B, consistently stable (*n* = 3 patients), Figure 4B, and divergent (*n* = 2 patients), Figure 4C. The two patients with consistent progression, L1 and L2, both carried exon 19 del and had increasing EV-RNA mutation burden that mirrored disease progression, Figure 4B. Both patients had no detectable EV-RNA mutations in their first visits, however, as their disease progressed, the mutation burden increased with each visit. L1 had increased size of lung nodules between visits 2 and 3, which correlated with the onset of detectable exon 19 del in their EV-RNA. Between visits 3 and 4, the size and number of lung nodules both increased, corresponding with a mutation burden increase of 223%. Similarly, patient L2 had progressing disease between visit 1 and 2, which was reflected by the onset of EV-RNA mutation detection. While the patient was clinically stable between visit 2 and 3, only a modest 40% increase in mutation burden was observed. L2’s disease further progressed after visit 3. Ultimately, both patients were placed in hospice shortly before their final time point in this study and are now both deceased.

The second category, comprised of patients L3–L5 who were consistently stable, all carried exon 19 del. They had sustained, clinically stable disease and showed a downward trend in their EV-RNA burden at nearly every time point, Figure 4B. L5 initially had brain metastases and high levels of EV-RNA, however, each subsequent time point demonstrated radiologically monitored resolution of brain metastases. This observation of sustained decreased EV-RNA burden is notably different than what was observed in L2, which showed a sustained increase in mutant EGFR burden, despite temporarily being classified as having clinically stable disease. All three patients with sustained clinical stability, L3–5, have been stable for 191, 182, and 204 days, respectively, since the final EV-RNA time point (Supplementary Figure 3).

In the last category, patients L9 and L10 each carried L858R and T790M and showed divergent clinical trajectories. L9 had undetectable levels of L858R and T790M EV-RNA at the initial visit and was clinically stable. However, as the patient progressed, EV-RNA burden of L858R and T790M increased between visits 3 and 4, 83 and 2460%, respectively (Figure 4C). At 87 days after the final blood draw, L9 has continued to show progression, and has brain metastasis (Figure 4). Conversely, L10 was progressing while on TKI therapy but did not have any detectable L858R or T790M EGFR burden. However, when the patient’s therapy was switched to chemotherapy (pemetrexed) and the disease stabilized, the L858R mutation became detectable and increased by 672% between visits 5 and 6 in EV-RNA, Figure 4C. This patient has continued to be clinically stable for 133 days since the final blood draw (Supplementary Figure 3).

To summarize the overall effectiveness of using EV-RNA as a correlate for disease outcome, the change in EV-RNA mutation burden was calculated between each sequential pair of time points. It was then determined whether the patient remained stable or had disease progression between the two time points in question. Promisingly, change in EV-RNA exon 19 del burden mirrored clinical trajectories at 12/14 time points across all five patients who had 2+ time points, with the definition of mirroring being shown in Figure 4A. Patients ranged from having 67% to 100% of time points where EV-RNA mutation burden mirrored disease trajectory, Figure 4D. When quantitatively compared, the change in exon 19 del EV-RNA transcripts (ΔEV-RNA) between time points is significantly lower for patients who are stable compared to progressing (*p*-value = 0.0059), Figure 4E. Conversely, the two patients with L858R/T790M mutations saw 33 and 40% mirrored time points for L858R EV-RNA and 67 and 60% mirroring for T790M EV-RNA, Figure 4F. It is important to note that for both point mutations, especially for T790M, the detection rate was low, therefore conclusions cannot be accurately drawn about the relationship between ΔEV-RNA burden and disease progression. As a comparison, absolute quantity of EV mutation burden was quantified between stable and progressing time points for both all mutations summed and each individual mutation. It was found that the absolute EV-RNA mutation burden is not significantly different between the stable and progressing time points, Supplementary Figure 6. Therefore, it is critical to use the change in EV-RNA mutation burden within each individual patient as a determinant of disease status.

## Discussion

Using ddPCR and western blots, EVs isolated from lung cancer patient plasma were analyzed for EGFR mutations. In this pilot study, the utility of EV-RNA and EV-protein is demonstrated to not only screen for the presence of mutations, but to dynamically monitor patient disease status. Mutant EV-RNA was detected in 9/10 patients, and for 6/7 patients who were longitudinally monitored, mutant EV-RNA burden mirrored clinical trajectory. Within patients who had exon 19 del, ΔEV-RNA mutation burden strongly indicated disease trajectory, demonstrating that a single time point may be insufficient to assess patient status. The power of liquid biopsies enabled multiple time points from each patient to be collected.

While evidence from this pilot study suggests the rate of increase in EV-RNA mutation burden may be linked with progression severity, larger studies are needed to investigate this. For the patients with exon 19 deletion, both patients who had consistent progressive disease succumbed to their disease and are now deceased. These patients had a similarly consistent increase in EV-RNA mutation burden. Conversely, the three patients with decreasing EV-RNA exon 19 del burden have remained clinically stable for an average of 192 ± 9 days after the final blood draw. These patients saw consistently decreasing EV-RNA mutation burden that mirrored their stable disease status. This was further highlighted by the finding that there is a significant difference in the exon 19 del ΔEV-RNA burden between time points between stable and progressing patients. These results indicate that changes in EV-RNA mutant burden may be an early indicator of clinical stability and perhaps even indicate disease progression before clinical monitoring methods.

Of the two patients with L858R/T790M mutations, one patient’s EV-RNA burden mirrored disease trajectory, while the other did not. L9 has had sustained progressive disease 87 days after the final blood draw. Conversely, L10, after switching from a TKI to chemotherapy between visits 4 and 5, has remained clinically stable for 133 days after the final blood draw, despite an increase in L858R EV-RNA. The change from TKI therapy to chemotherapy during this study could impact the utility of targetable mutations carried in EV-RNA for patient monitoring. Without the use of TKIs specifically targeting mutant EGFR, an increase mutant burden may not indicate treatment resistance in the tumor. Therefore, while EV-RNA may mirror disease trajectory for patients receiving targeted therapy, this may not extend to patients receiving other treatment types, such as chemotherapy and further studies are needed to investigate this.

Additional studies with larger cohorts are needed to validate these findings, however, this study presents initial evidence that increase in EV-RNA indicates progression for patients receiving targeted therapy. Future studies are needed to determine if EV-RNA can be used to detect progression prior to current techniques. Of interest, L6, only had two time points collected but saw an upward trend in their burden despite being clinically stable thus far, Supplementary Table 2. The preliminary findings presented here warrant a recommendation that the clinical trajectory of this patient should closely be monitored for indicators of disease progression.

While EV-protein was detected in samples from four patients, there was not an observed correlation between EV-protein burden and EV-RNA burden and there were too few patients to make assessments of correlation to disease. This could be due to the differences in EV packing of RNA cargo to protein cargo, which are largely poorly understood, especially in the ever-changing physiological states found during cancer immunotherapies. Beyond EV packaging differences, interestingly, previous studies in cell lines have shown that when exposed to TKIs, EGFR mutations result in differential protein stability compared to wildtype (Ray et al., 2016); Treatment with the TKI erlotinib led to protein degradation in a mutant dependent manner, without significantly changing the transcriptomic expression. Additionally, osimertinib, the primary TKI the patients in this study were receiving, has been suggested to reduce protein stability in both wildtype and T790M mutant EGFR. The decreased stability of EGFR protein due to TKI treatment could lead to increased cellular protein turnover thereby either (1) reducing the amount of protein packaged into EVs or (2) leading to increased degradation of either cellular protein or EV-protein, both of which would reduce the quality of the proteins found in EVs, as seen in this study (Ray et al., 2016). To answer this question, future studies could investigate the ratio of EGFR wildtype to EGFR mutated protein found within specifically tumor derived EVs through treatment to elucidate the stability of these mutated proteins. Further studies will need to be performed to elucidate information about the mutated protein found in EVs.

Liquid biopsies hold the potential to address spatial heterogeneity and longitudinal monitoring limitations. However, the only FDA-approved liquid biopsy test, the Cobas v2, for mutation detection, relies on ctDNA. While capable at detecting the presence of new mutations, this test is still currently considered a “rule in” test, with the recommendation of a tissue biopsy to confirm a mutant negative result (Odogwu et al., 2018). Compared to ctDNA, EVs demonstrated a more robust detection of exon 19 del and L858R point mutation, and similar detection rates for the point mutation T790M, Figures 4E,F. To avoid splitting the sample or using pre-amplification steps, this study screened solely for EGFR mutations detected by tumor biopsy, therefore future studies are needed to expand further to screening for mutations in EV-RNA not originally detected by tumor biopsy. By instead using EVs, liquid biopsy mutation screening has the potential to advance beyond the current “rule in” test.

This work lays the groundwork for future studies to establish the utility of mutations found in EV cargo for patient care. In this novel proof of concept study, EVs were screened for previously identified EGFR mutations carried by each patient. Changes in EV-RNA correlated with disease trajectory; however, the clinical implications of EV-protein remain unclear. The utility of EV mutation monitoring warrants further investigations across additional mutations and cancer types. Further, the dual analysis of EV-derived cargo has the potential to go beyond monitoring and be used in lieu of a tumor biopsy for non-invasive screening for both sensitizing and resistance mutations in EGFR across a patient’s treatment course. This minimally invasive approach could be integrated into the standard of care enable more rapid identification of treatment resistance and allow for timely treatment changes, overall improving patient care.

## Materials and Methods

### Cell Culture

H1975, H3255, and H1650 cells were grown in RPMI-1640 (Gibco) supplemented with 10% fetal bovine serum (FBS) (Sigma-Aldrich) and 1% Antibiotic-antimycotic (Gibco). Cells were grown to 80% confluence before subculturing using 0.05% Trypsin-EDTA (Gibco). To prepare extracellular vesicles, cells were seeded at 3,000,000 cells/100 mm dish (Sarstedt) in complete media. 24 h after seeding, cells were washed three times with phosphate-buffered saline (PBS) pH 7.4 (Gibco) and incubated for 72 h in serum-free RPMI-1640 media (Gibco). Cell culture media (CCM) was centrifuged at 2,000 × *g* for 15 min and frozen at −20°C.

### Patient Enrollment

All blood was collected following IRB (HUM00119934) approval, and all patients gave their informed, written consent to participate in the study. All patients had metastatic lung adenocarcinoma. The cohort of patients in this study had known EGFR mutations.

### Blood Preparation

Plasma was prepared for EV isolation using one of the following three methods and was stored in the −80°C freezer until use.

*Plasma prep 1 (Ficoll):* Whole blood was collected in EDTA tubes. Samples were prepared using Ficoll-Paque^TM^ PLUS (GE Healthcare) following the manufacturer’s protocol. The plasma and leukocyte layers were collected for CTC isolation and effluents were centrifuged following the plasma prep 3 protocol.

*Plasma prep 2 (Dextran):* Whole blood was collected in EDTA tubes. 1 mL of 6% dextran solution (w/v) was mixed into 5 mL whole blood. The sample sat 1–1.5 h at room temperature to allow the red blood cells sedimentation. The supernatant was collected for CTC isolation and the effluent was centrifuged following the plasma prep 3 protocol.

*Plasma prep 3:* Whole blood was collected in EDTA tubes was centrifuged at 2,000 × *g* for 15 min at room temperature. The plasma supernatant was collected and frozen at −20°C for up to 30 days.

### Extracellular Vesicle Isolation Using Differential and Ultracentrifugation

Plasma prep or CCM was centrifuged at 12,000 × *g* for 20 min to remove cellular debris. The supernatant was then ultracentrifuged at 100,000 × *g* for 90 min to pellet the EVs using 36 mL Polyethylene terephthalate (PET) tubes (Thermo Fisher Scientific). Excess tube volume was filled with sterile PBS pH 7.4 (Gibco). The extracellular vesicle-pellet was washed with PBS and centrifuged at 100,000 × *g* for 90 min. Extracellular vesicles were suspended in 100 μL PBS pH 7.4 or RIPA with protease inhibitor cocktail (Thermo Fisher Scientific) and frozen at −20°C. EVs stored in PBS were then used for nanoparticle tracking analysis along with RNA extraction and characterization, while EVs in RIPA were used for western blot analysis.

### Extracellular Vesicle Quantification

Following isolation, extracellular vesicles were quantified for size and concentration using NTA using Malvern’s NanoSight. Quantification was performed using five-30 s runs at a flow rate of 20 using the brightness setting of 15. The camera detection was set to a level of 4 for all runs. Runs were then averaged with the average and standard deviation between the runs being reported.

### RNA Extraction and Reverse Transcription

Ultracentrifuged extracellular vesicles were lysed using TRIzol^TM^ Reagent (TRIzol) (Invitrogen) at a 1:10 ratio of extracellular vesicle suspension to TRIzol and incubated at room temperature for 5 min. A 1:5 ratio of chloroform (Sigma-Aldrich) to TRIzol was added and briefly vortexed to mix, then incubated for 2–3 min at room temperature. The sample was centrifuged at 12,000 × *g* for 15 min. The aqueous phase was collected and mixed in a 1:1 ratio with 70% ethanol (Sigma-Aldrich). Total RNA was purified using the Norgen Single Cell RNA isolation kit (Norgen Biotek Corp.). cDNA was prepared using SuperScript IV VILO Master Mix with ezDNase Enzyme (Invitrogen) following the manufacturer’s protocol. All purified RNA and cDNA products were handled in a PCR workstation to prevent contamination.

### RT-qPCR

Twenty microliter TaqMan^TM^ gene expression PCR reactions were prepared using TaqMan^TM^ Fast Advanced Master Mix (Applied Biosystems) in 96-well MicroAmp Fast Optical Plates (Applied Biosystems) and processed on a QuantStudio 3 (Applied Biosystems) using fast cycling conditions. Each mRNA: sample pair was analyzed in technical triplicates.

**Table:** 

**TaqMan^**TM**^ gene expression assay IDs**	
Gene	Assay ID
ACTB	Hs01060665_g1
GAPDH	Hs03929097_g1

### Cell-Free DNA Extraction

Cell-free DNA (cfDNA) was isolated from the plasma using the QIAamp Circulating Nucleic Acid Kit (Qiagen) following the manufacturer’s processing protocol. cfDNA was eluted into 15 μL for ddPCR mutation detection.

### Mutation Detection by Droplet Digital PCR

Epidermal growth factor receptor mutations were identified by using RainDrop^TM^ ddPCR (RainDance Technologies). In brief 25 μL reactions were prepared using TaqMan^TM^ SNP Assay (Life Technologies), 2x TaqMan Genotyping Master Mix (Applied Biosystems), and droplet stabilizer (RainDance Technologies). Maximum cDNA was loaded into each dPCR reaction. The PCR reaction was loaded onto the Source Chip (RainDance Technologies) to for droplet generation and collected into an 8-tube PCR strip (Axygen). The PCR tubes were transferred to the thermocycler for 45 rounds of PCR amplification (Bio-Rad). The PCR tubes, containing the samples, were then transferred onto the Sense Machine (RainDance Technologies) where the fluorescence intensity of each droplet was measured.

For the point mutations, L858R and T790M, mutations were considered present based on the detection of one or more positive droplets within the pre-established gates based on positive EV-RNA controls. For exon 19 del, deletions were considered present based on the detection of one or more positive droplets above the threshold. A threshold for detection was determined based on the number of false positive droplets detected using EV-RNA negative controls. The maximum number of false positive droplets detected in any negative control (16 droplets) was used as the threshold for detection. All presented data is represented as the threshold subtracted from the total number of mutant positive droplets counted and any further normalization specified in the respective figure.

**Table:** 

**TaqMan^**TM**^ EGFR mutation detection assay IDs**	
**Gene**	**Assay ID**

L858R	AHRSRSV
T790M	AHRSROS
Exon 19 deletion	Hs00000228_mu

### Protein Extraction, Quantification, Normalization

Extracellular vesicles were isolated from ultracentrifugation into 150 μL RIPA buffer (Thermo Fisher Scientific, cat #89900), protein concentration was measured by Micro BCA^TM^ Protein Assay Kit (Thermo Fisher Scientific cat #23235). Western blot loading was normalized by using 5 mL blood volume for extracellular vesicle isolation, loading the maximum protein (37.5 μL) in each lane, and using Bio-Rad’s Stain Free gels to allow normalization. Briefly, the protein was separated at 250 V for 30 min. Semi-dry transfer was then performed using Trans-Blot Turbo Transfer System (Bio-Rad) to a high fluorescence PVFD membrane (Bio-Rad, cat #1620261). The membrane was imaged using Bio-Rad’s ChemiDoc to quantify total protein per lane. The membrane was blocked and incubated overnight with primary antibody in 5 mL of 5% bovine serum albumin (Sigma-Aldrich) in Tris Buffered Saline (TBS) (Bio-Rad) with 1% Tween 20 (Sigma-Aldrich) (TBST). The membrane was then washed thoroughly before incubating with HRP-secondary antibody in 3% non-fat milk in TBST for 90 min followed again by additional washes. Measurement was performed using SuperSignal^TM^ West Pico PLUS Chemiluminescent Substrate (Bio-Rad, Cat #34579) and SuperSignal West Femto (Thermo Fisher Scientific, cat #34096) and imaged on the ChemiDoc.

**Table:** 

**Antibodies used for Western Blot**		
**Target**	**Dilution**	**Catalog number (Cell Signaling)**

CD9	1:1000	#13174
ACTB	1:1000	#4970
GAPDH	1:1000	#5174S
Calnexin	1:1000	#2679
EGF Receptor L858R Mutant Specific	1:1000	#3197
EGF Receptor exon 19 E746-A750 del specific	1:1000	#2085
Anti-rabbit IgG, HRP-linked Antibody	1:1500	#7074S

Normalization was performed following Bio-Rad’s Stain Free Gel analysis protocols. Briefly, following protein separation and transfer, the blot is imaged using Bio-Rad’s Stain Free Blot imaging setting to capture the total protein per lane. Using Bio-Rad’s Image Lab 6.0.1 software, the total protein in each lane is compared and a normalization coefficient determined. After blotting for specific proteins, each band is compared to the total protein of the lane, adjusted using the normalization coefficient, and quantified as a Normalized Protein Intensity.

### Statistical Analysis

All analysis was performed in GraphPad Prism V9. *P* values were calculated using unpaired, two tailed, *t*-tests.

## Data Availability Statement

The original contributions presented in the study are included in the article/Supplementary Material, further inquiries can be directed to the corresponding author.

## Ethics Statement

The studies involving human participants were reviewed and approved by the University of Michigan Institutional Review Board #HUM00119934. The patients/participants provided their written informed consent to participate in this study.

## Author Contributions

EPu and SO designed the study, interpreted the data, and co-wrote the manuscript. EPu, AR, NC, and CS developed the protocols and performed the western blots and NTA. SO and EPr performed the ddPCR experiments. T-WL performed the SEM. MZ reserved the CTC isolation effluent for use in this study. NR and SN oversaw the study, managed ethical patient enrollment and sample collection, and revised the manuscript. All authors contributed to the article and approved the submitted version.

## Conflict of Interest

The authors declare that the research was conducted in the absence of any commercial or financial relationships that could be construed as a potential conflict of interest.

## Publisher’s Note

All claims expressed in this article are solely those of the authors and do not necessarily represent those of their affiliated organizations, or those of the publisher, the editors and the reviewers. Any product that may be evaluated in this article, or claim that may be made by its manufacturer, is not guaranteed or endorsed by the publisher.
