# Supplementary material for: Epidermal Growth Factor Receptor Mutations Carried in Extracellular Vesicle-Derived Cargo Mirror Disease Status in Metastatic Non-small Cell Lung Cancer
Source: Front Cell Dev Biol. 2021 Oct 6;9:724389. doi: 10.3389/fcell.2021.724389 (PMC8526851; doi:10.3389/fcell.2021.724389)

***Supplementary Material***

# Supplementary Figures and Tables


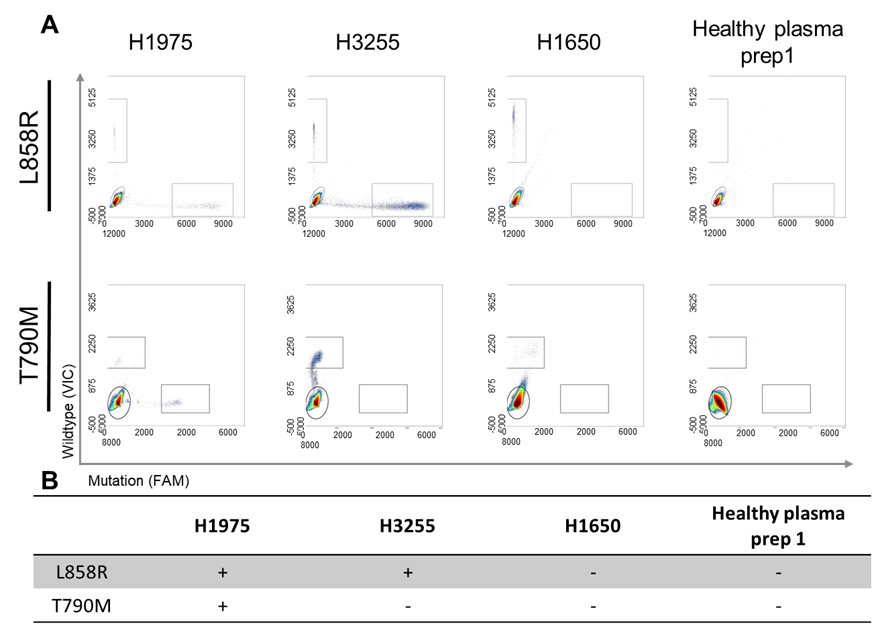


**Supplementary Figure 1.** **EGFR point mutations, L858R and T790M, detected in lung cancer cell line derived EV-RNA. A.** Representative ddPCR plots for EGFR L858R (top) and T790M (bottom) from EV-RNA generated using control lung cancer cell line derived EVs and healthy control EVs from plasma prep 1. The mutation signal is detected in the FAM channel (x-axis), wildtype signal is detected in the VIC channel (y-axis) and empty droplets are shown in the bottom left of each plot. **B.** Table of EGFR L858R and T790M point mutation status in lung cancer cell lines and healthy donors.


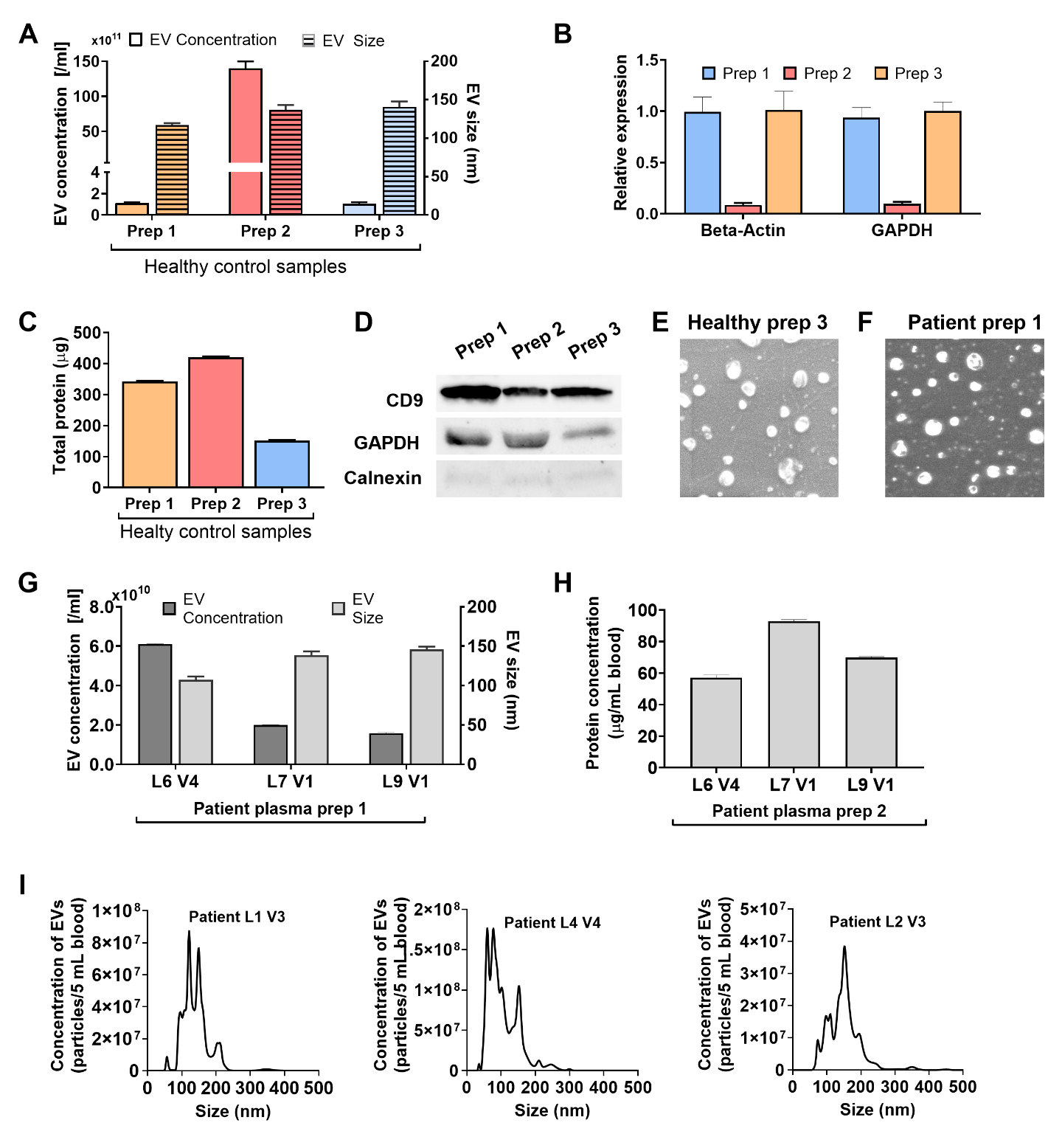
**Supplemental Figure 2. Comparison of plasma preparations for EV analysis.**

EVs were isolated from the effluent material generated in a tangential circulating tumor cell (CTC) characterization study. 15mL of blood from a healthy donor was divided and processed using 5mL of blood using each of the three plasma generation methods: Ficoll RBC removal followed by centrifugation (prep 1), dextran RBC removal followed by centrifugation (prep 2) or centrifugation alone (prep 3). For all reported values, error bars are the standard deviation of 3 technical replicates.

**A.** EV size and concentration of the three prep methods was measured using NTA and the size of the EVs did not vary with isolation method. All yielded vesicles were within the standard range for EVs (50-160 nm). The concentration has been normalized to starting blood volume, reported values are per mL of original blood volume. The measured concentration of the EVs isolated using prep 2 was dramatically higher than the concentration from prep methods 1 or 3.

**B.** The relative abundance of house-keeping genes, ACTB and GAPDH, was measured using RT-qPCR. Using prep 3 EVs as the baseline for comparison, prep 1 EVs had similar levels of the two genes, whereas prep 2 EVs had less than 10% comparatively. Because the detected levels of housekeeping genes were similar for preps 1 and 3, prep 1 samples were used for EV-RNA characterization.

**C-D.** EV-protein characterization was performed using microBCA and western blot. There was slightly higher protein yield from prep 2 than prep 1, and both were higher than prep 3 (**C**)**.** Western blot analysis of the three plasma prep methods showed similar expression of the following proteins: EV marker CD9, housekeeping gene GAPDH, and contamination marker calnexin (**D**)**.**

**E-F.** Scanning electron microscopy images of EVs isolated from a healthy control prep 3 (**E**) and a patient prep 1 (**F**) to demonstrate successful EV isolation

**G.** Prep 1 from 3 NSCLC patient blood samples were processed either (1) directly into PBS for NTA quantification or (2) into RIPA lysis buffer for protein analysis. Three plasma prep 1 samples were measured using NTA and were found to have similar size, with an average of 130 ± 17nm across the three samples, and an average concentration of 3x10^10^ ± 2x10^10^.

**H.** From the same patients and time points as **G**, the prep 2 samples were measured for EV-protein quantity using microBCA with an average across the three samples of 73 ± 18 µg/mL of blood in 150 µL of protein lysate from 5mL of blood**.**

**I.** Representative Nanosight NTA graphs are shown for prep 2 from 3 patients, L1, L2, and L4. These graphs show the size of EVs to be predominantly under 200nm.

**Supplemental Figure 3. Clinical data timeline mapped to the mutant EV-RNA at each visit.**
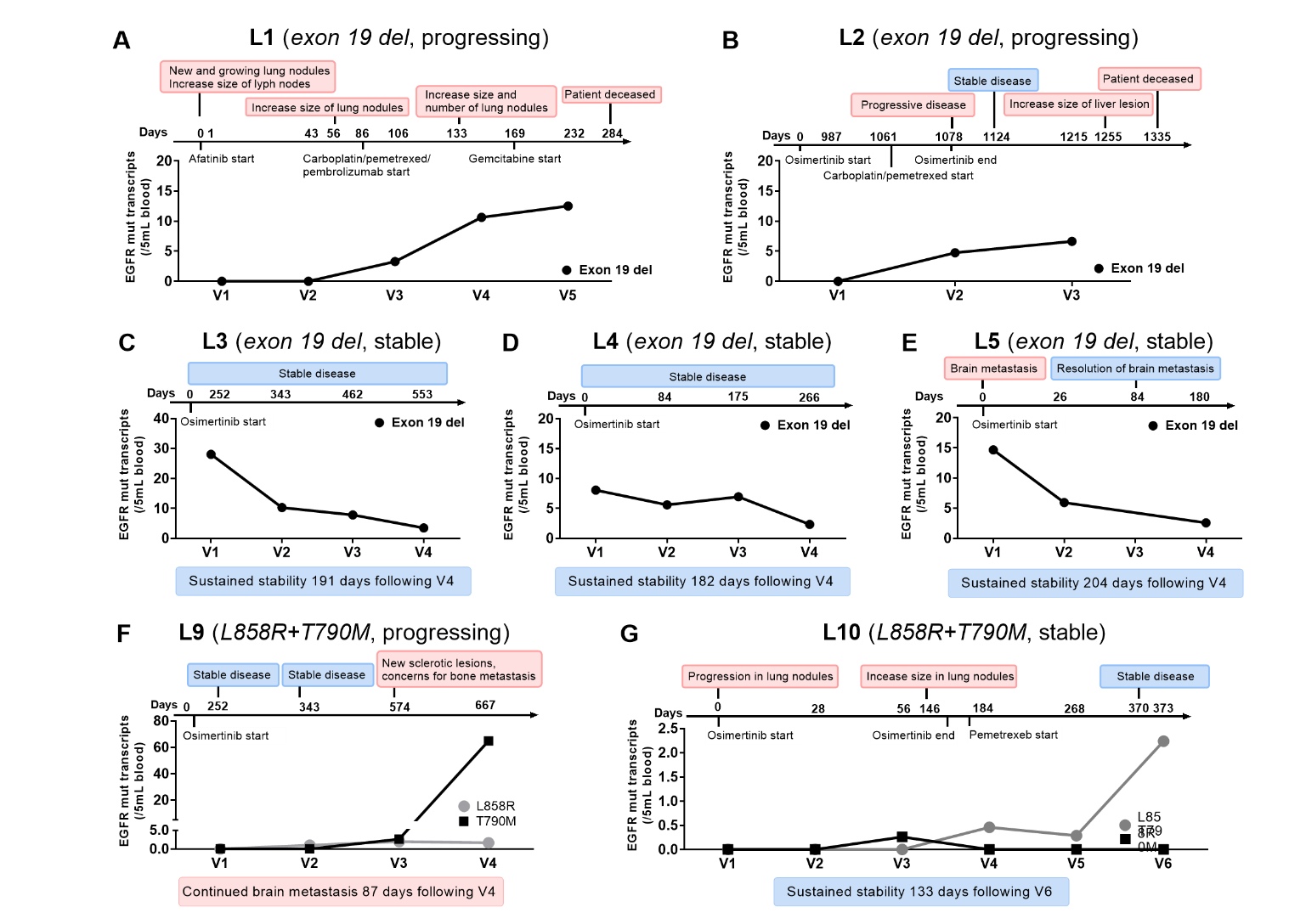


**A-G.** Normalized droplet counts for EGFR mutations in EV-RNA using ddPCR. A timeline with each patient’s clinical data is displayed in days from start of treatment. Clinical data showing disease progression is shown in red text boxes, while stable disease is shown blue text boxes.


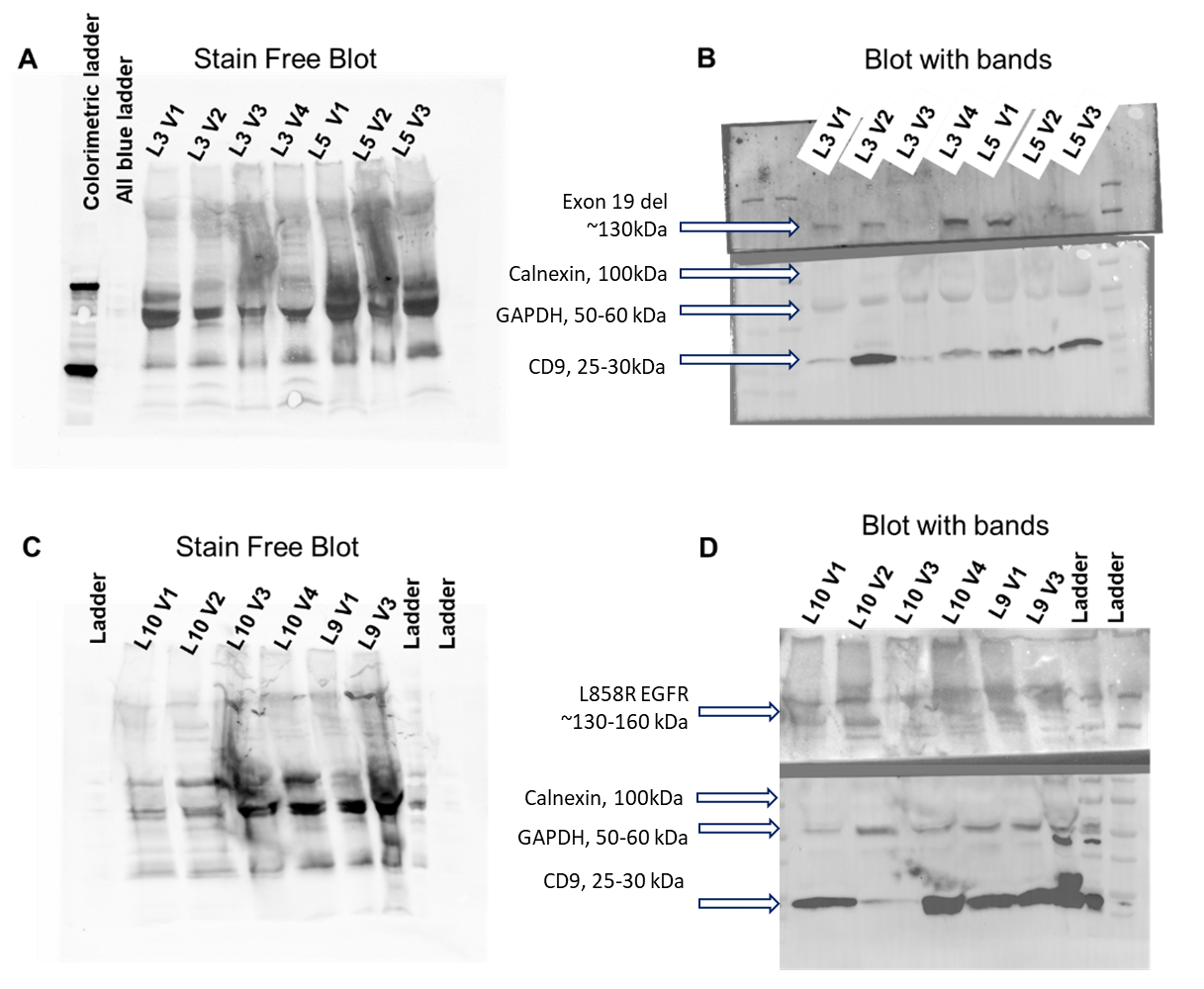


**Supplemental Figure 4. Full western blot images for patient EV protein samples**

**A, C.** Stain Free Blot images showing the total protein per sample, used to perform quantification of specific bands. Each lane represents the total protein per sample, with an increase in pixels in a certain area demonstrating an increase in protein concentration at that molecular weight. These are performed using BioRad’s StainFree Gels, 4-20%.

**B, D.** Bands for 4 proteins of interest. The blots were cut in half horizontally to split into ~120-250kDa in the top half, and 10-110kDa in the bottom half, as determined using ladders on both edges of the blots. The top image in each part demonstrates an EGFR mutation: either exon 19 del (**B)** or L858R (**D).** Using the bottom half of the blots, the samples were additionally tested for calnexin (MW ~90-100kDA), GAPDH (~50-70kDA), and CD9 (~25-35kDA). Calnexin, a cellular contamination marker, is negative or minimally expressed in all samples. GAPDH has varying expression levels, indicating the need for total protein quantification. CD9 is expressed in all samples, at varying levels.


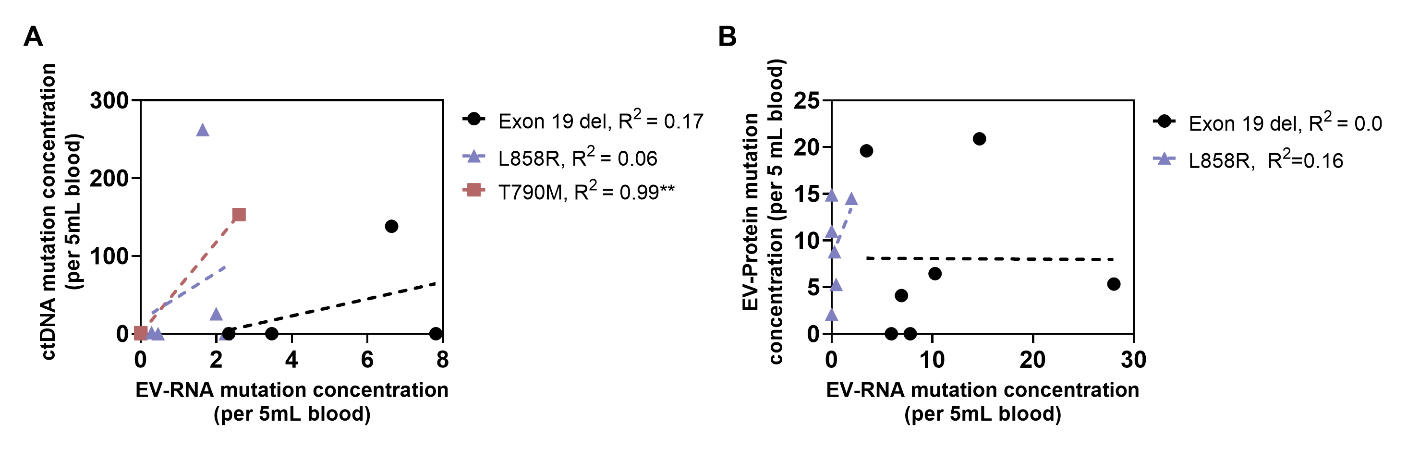
**Supplemental Figure 5. Correlation of mutation burden between EV-RNA, EV-Protein, and ctDNA.**

**A-B.** Correlation between **A.** EV-RNA and ctDNA. and **B.** EV-RNA and EV-Protein by plotting the signal of the mutation found in 5mL of blood for each of the biomarkers (EV-RNA, ctDNA, EV-Protein) for each comparative timepoint. The R^2^ was calculated using a linear regression and each point represents one time point for one patient. **, although the R^2^ is approximately 1, this is because of the singular outlying data point and as such should not be considered significant.


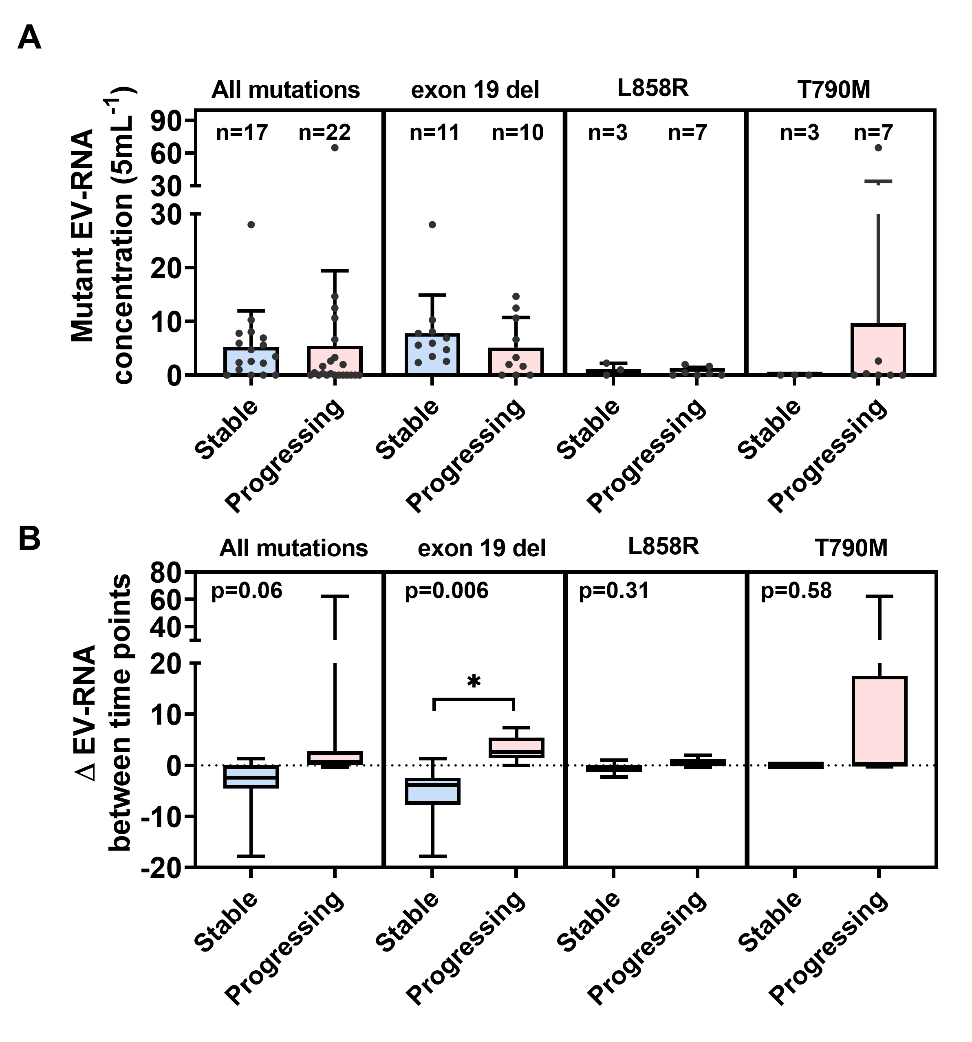


**Supplemental Figure 6. Comparison of absolute quantity of EV-RNA mutation burden at individual timepoints and the change in EV-RNA mutation burden between timepoints for grouped stable and progressing timepoints.**

**A.** Mutant EV-RNA concentration per 5 mL of blood for individual time points classified as either stable or progressing based on clinical assessment. This analysis is performed for all mutations grouped and each mutation separately.

**B.** The change, Δ, in EGFR EV-RNA mutation burden is calculated between every pair of two sequential timepoints. The change between these timepoints is then classified as either stable or progressing. * < 0.05.

**Supplemental Table 1. Patient demographics and clinical information.**

| **Patient** | **Gender** | **Age** | **Race** | **Stage** | **Node status** | **Tumor** | | **EGFR Mutation** | **Treatments Used** | **Current patient status** |
| --- | --- | --- | --- | --- | --- | --- | --- | --- | --- | --- |
| L1 | Female | 45 | White | IV | N3 | T4 | Exon 19 deletion,  w/ PDL1 10% | | Osimertinib, carboplatin + pemetrexed + pembrolizumab, gemcitabine | Deceased |
| L2 | Male | 72 | White | IV | NA | NA | Exon 19 deletion, T790M | | Osimertinib, carboplatin + pemetrexed | Deceased |
| L3 | Male | 62 | White | IV | N2 | T3 | Exon 19 deletion | | Osimertinib | Stable |
| L4 | Male | 70 | White | IV | N0 | T2a | Exon 19 deletion | | Osimertinib | Stable |
| L5 | Male | 66 | White | IV | N0 | T1a | Exon 19 deletion | | Osimertinib | Stable |
| L6 | Female | 61 | White | IV | N0 | T2 | Exon 19 deletion | | Erlotinib | Stable |
| L7 | Female | 70 | White | IV | N3 | T1a | Exon 19 deletion | | Osimertinib | Stable |
| L8 | Female | 82 | White | IV | NA | NA | Exon 19 deletion | | Osimertinib | Stable |
| L9 | Female | 61 | White | IV | N3 | T2 | L858R, T790M | | Osimertinib | Progressing |
| L10 | Female | 58 | White | IV | N0 | T2 | L858R, T790M | | Osimertinib and pemetrexed | Stable |

**Supplemental Table 2. Patient EGFR mutation ddPCR and extracellular vesicle characterization.**


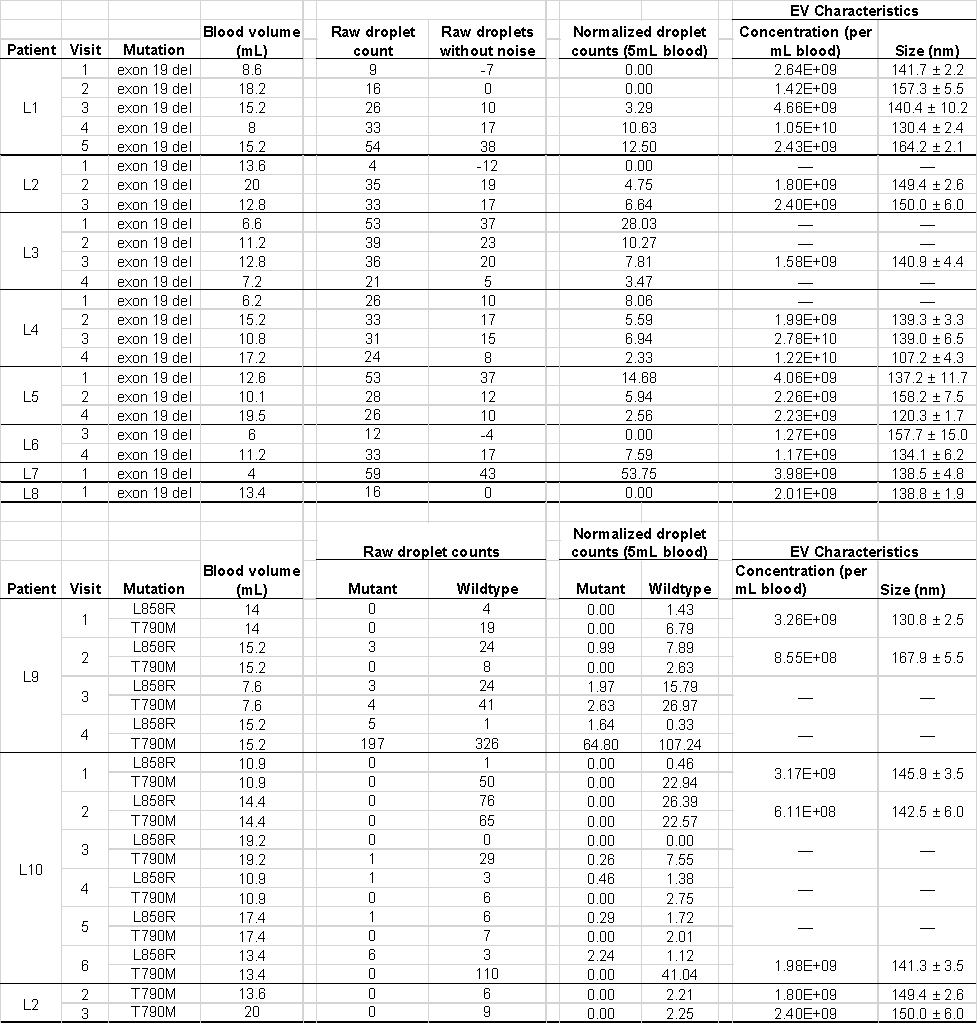

Supplement: Supplementary file 1 [file Data_Sheet_1.docx]
